# Genomics in the renal clinic - translating nephrogenetics for clinical practice

**DOI:** 10.1186/s40246-015-0035-1

**Published:** 2015-06-24

**Authors:** Andrew Mallett, Christopher Corney, Hugh McCarthy, Stephen I. Alexander, Helen Healy

**Affiliations:** Kidney Health Service & Conjoint Kidney Research Laboratory, Royal Brisbane and Women’s Hospital, Butterfield Street, Herston, Brisbane, Qld 4029 Australia; Centre for Kidney Disease Research, CKD.QLD and Centre for Chronic Disease, School of Medicine, The University of Queensland, Brisbane, Australia; Centre for Rare Diseases Research, Institute for Molecular Bioscience, The University of Queensland, Brisbane, Australia; Department of Paediatric Nephrology, Children’s Hospital at Westmead, Sydney, Australia; Centre for Kidney Research, University of Sydney, Sydney, Australia; Discipline of Paediatrics and Child Health, University of Sydney, Sydney, Australia

**Keywords:** Nephrology, Genomics, Translation

## Abstract

Genetic Renal Disease (GRD) presents to mainstream clinicians as a mixture of kidney-specific as well as multi-organ entities, many with highly variable phenotype-genotype relationships. The rapid increase in knowledge and reduced cost of sequencing translate to new and additional approaches to clinical care. Specifically, genomic technologies to test for known genes, the development of pathways to research potential new genes and the collection of registry data on patients with mutations allow better prediction of outcomes. The aim of such approaches is to maximise personal and health-system utility from genomics for those affected by nephrogenetic disorders.

## Introduction

Chronic kidney disease (CKD) in paediatric and adult populations produces a significant burden of morbidity and mortality [1] and frequently has a genetic aetiology [2]. However, Genetic Renal Disease (GRD) itself represents a heterogeneous and poorly defined group of, often rare, disorders. GRD is the cause for 10 % of kidney disease in adult renal practice [3] and up to 10–15 % and 50 %, respectively, of adults and children commencing dialysis [4]. The emergence of genomic technologies presents opportunities for better delineation of these conditions and for further gene discovery, thus overcoming genetics exceptionalism to enable optimised clinical care and providing the opportunity to develop targeted therapies. To realise this potential for patients, families and their physicians, managing increasing volumes and complexity of genetic and clinical information requires a multifaceted novel approach.

## Main text

Over the past decade, genomics and genomic technologies have resulted in a greater integration of genetics into healthcare [5]. Whilst sporadic renal genetics clinics (RGCs) have been established, there is little data on the specific models employed. RGCs attempt to provide a better way for patients and families with rare nephrogenetic diseases in accessing appropriate diagnostics, useful disease-specific information, genetic counselling, and highly specialised therapies in a centralised patient-focussed approach.

Currently, these clinics are located in tertiary hospitals, accessible to patients with rare/genetic diseases, with specialised nephrologists with genetic training and clinical geneticists, genetic counsellors, specialised nurses and allied health with whom to operate combined clinics. Additionally, RGCs need close contact with diagnostic laboratories and scientists. Such multidisciplinary clinics are increasingly necessary for delivering care in rare genetic disease [6]. Examples include ophthalmology, cardiology, oncology, hepatology, neurology and otolaryngology. These innovative clinical models have been encouraged as part of health service delivery reorganisation to deliver more patient-focussed care [7]. Successful models share in common an interaction between clinical geneticists, sub-specialists, genetic counsellors, speciality nursing staff and other allied health staff with ongoing collaborations with molecular geneticists and scientists. One challenge is a general lack of sub-specialised professional staff; however, targeted training and education are likely to overcome this in the medium-long term. Another challenge is patient transition from paediatric to adult healthcare, which may be remedied by active involvement of paediatric nephrologists within the RGC in order to provide lifespan and whole family care. A third potential challenge is the pitfalls associated with genetic testing, including pre-symptomatic testing in at-risk individuals, prenatal diagnostics, non-paternity/maternity and medically actionable incidental findings in whole exomic or genomic sequencing. Within the RGC, patient consultation with clinical geneticists and genetic counsellors is ideally placed as a standard-of-care to minimise such risks whilst maximising patient benefit and support. Further, additional specialists are required for GRDs with extra-renal involvement, such as oncologists and endocrinologists for *WT1* disorders, metabolic physicians and neurologists for mitochondrial disorders and hepatologists and ophthalmologists for ciliopathies. Supportive medical administrators and molecular geneticists enhance the model further. An RGC should be no different, with natural implications for broader patient management such as medical interventions, therapies and genetic counselling.

GRDs are phenotypically diverse and attributed to growing numbers of known genes, with increasingly complex phenotype-genotype relationships. Genomics enables rapid diagnosis of mutations in known nephrogenes. This includes very efficient sequencing of many known genes simultaneously and effectively for ciliopathies [8], podocytopathies and glomerular disease [9, 10], complement dysregulopathies [11], congenital abnormalities of the kidney and urinary tract (CAKUT) [12], tubular disease and nephrocalcinosis/nephrolithiasis [13]. This may be via whole exome or genome sequencing, or targeted and prioritised gene panel screening of these. For several diagnoses, precise genetic diagnosis drives therapeutic decisions. Examples of this include steroid-resistant nephrotic syndrome where therapies with significant side-effect profiles are unlikely to be effective, Alport syndrome in which the early instigation of renin-angiotensin system blockade may delay renal dysfunction progression and atypical haemolytic uraemic syndrome in which some genotypes (*CFH*) are associated with more pressing requirements for eculizumab or combined liver-kidney transplantation whilst others (*C5*, *DGKE*, *MMACHC*) may confer altered responses to medical therapy. There are also indirect and surprising findings. For instance, where 12 genes are responsible for only 6 % of unselected paediatric CAKUT cases [12], 14 genes are causative for 15 % of similarly unselected nephrolithiasis/nephrocalcinosis [13]. Intriguingly, CAKUT is a common paediatric nephropathy of potentially and well-recognised genetic aetiology, where nephrolithiasis/nephrocalcinosis is a common adult renal presentation of potentially though “rare” genetic aetiology. Genomics is altering prevailing concepts of kidney disease, including diagnosis and therapy, whilst translating to changes in nephrological practice.

Amongst other applications, genomics has also opened the frontier to discovery of undescribed causative genes. A genomic approach provides a research pathway to discovery in those with individually rare undiagnosed GRDs. For example, the identification of a specific *LMX1B* mutation associated with a renal-limited rather than multisystem form of Nail-Patella syndrome [14] challenges classical phenotype-genotype relationships. Other examples include the findings of podocyte-related gene mutations in some patients with histological membranoproliferative glomerulonephritis [10], mutations in a ciliary gene (*TTC21B*) causing focal segmental glomerulosclerosis [15] and mutations in a recently described gene (*DGKE)* resulting in atypical haemolytic uraemic syndrome [16], membranoproliferative glomerulonephritis [17] and steroid-resistant nephrotic syndrome [10]. Similarly, the discovery of mutations in DNA damage repair genes (*ZNF423*; *CEP164*) causing nephronophthisis [18] expands pathophysiological understanding. Such advances have potential implications for future directed approaches to treatment, as these are likely to be underpinned by accurate understanding of disease pathogenesis. The autosomal recessive spectrum of nephronophthisis and related ciliopathies continues to present promising opportunity for gene discovery utilising genomic technologies [19–21], a necessary prequel to translational proteomics [22].

There are many specific examples of the rapidly expanding diagnostic success of massively parallel sequencing (MPS). An instance has been Autosomal Dominant Tubulointerstitial Kidney Disease, a subset of which was chronically refractory to MPS [23] though subsequently found to be due to specific *MUC1* mutations. Further, accurate genomic sequencing is notoriously difficult for Autosomal Dominant Polycystic Kidney Disease (*PKD1* and *PKD2* mutations) due to homology with pseudogenes [24]. This assumes significance as this is the most common potentially lethal monogenic inherited disease amongst humans. Whilst genetic approaches have overcome these obstacles for diagnostic purposes [25, 26], they highlight that genomic studies may not always adequately resolve specific GRDs and that one needs to be cognisant of technological limitations, including copy number variations [27], read depth, coverage of regions of interest, a relative lack of bioinformaticians in the healthcare system and incomplete knowledge. The utilisation of family-based bioinformatics, such as trios, in genomic studies for gene discovery and diagnosis [28] appears crucial, especially when a complex clinical phenotype has not been appreciated [29].

For many GRDs, only a fraction of the genetic causes is known. There are three major issues that dominate the application of new genetics to kidney disease. Firstly, the increasing heterogeneity of phenotypes linked to genes, secondly, many clinical phenotypes have numerous differing genetic and undiagnosed causes, and thirdly, frequent extra-renal organ involvement in GRDs. Whilst many genetic causes can be identified, variants of uncertain significance may be found. In such cases, intra-family segregation analysis or functional study of such variants in pre-clinical non-human, fibroblast, or autologous self-organising renal organoid model systems [30] may be utilised to clarify variant significance. Families and individuals in whom a genetic cause seems likely can then be directed into discovery programmes to illuminate their disease. Therefore, this increasingly variable and rapidly changing field now needs a co-ordinated team approach linking patients, clinicians and scientists. This may have health economic benefits within the context of increased uptake of emerging genomic technologies in the RGC, whilst due caution is required with regard to local legislative, regulatory, health funding and insurance environments.

A recent Norwegian population study suggests that the magnitude of undiagnosed GRD may be as large as diagnosed GRD [31]. These findings highlight the importance of epidemiological and registry studies as an adjunct to genomic diagnostics or discovery studies. Epidemiological investigation is critical to describe and target nephrogenetic conditions for investigation utilising genomic technologies. Particularly to prevent confusion when disease or genotype frequency estimations reported for single-centre or highly selected cohorts [32] have resulted in over-estimation. This becomes apparent when large multicenter cohorts are further examined [12] or when interrogation of such cohorts for highly defined phenotypes is undertaken [33].

## Conclusions

In conclusion, an initial step for nephrogenetic clinical translation is to progress a conversation defining patient-centric priorities within a changing context of clinical care and genomic technologies. We postulate that this involves reconceptualising clinical interfaces, applying such genomics for clinical diagnosis of known nephrogenes, utilising genomics in research to discover new genetic aetiologies and furthering epidemiological investigation of GRD. The RGC is a proposed multidisciplinary construct potentially capable of incorporating and actioning such changes. The aim of such an approach is to maximise personal and health-system benefit from genomics for those affected by nephrogenetic disorders. This approach reflects the increasing likelihood for the explanation of the majority of inheritable causes for kidney disease and, in the future, defined and effective treatments.

## Abbreviations

CAKUT: Congenital abnormalities of the kidney and urinary tract
GRD: Genetic Renal Disease
MPS: Massively parallel sequencing
RGC: Renal genetics clinic
